# Pro-Adrenomedullin predicts 10-year all-cause mortality in community-dwelling patients: a prospective cohort study

**DOI:** 10.1186/s12872-017-0605-3

**Published:** 2017-07-04

**Authors:** Jonas Odermatt, Marc Meili, Lara Hersberger, Rebekka Bolliger, Mirjam Christ-Crain, Matthias Briel, Heiner C. Bucher, Beat Mueller, Philipp Schuetz

**Affiliations:** 1 Department of Endocrinology, Diabetology and Metabolism, Medical University Clinic, Kantonsspital Aarau and University of Basel, Switzerland, Tellstrasse, CH-5001 Aarau, Switzerland; 2 Department of Endocrinology, Diabetology and Metabolism, Department of Clinical Research, University Hospital Basel, Basel, Switzerland; 3 Department of Clinical Research, Basel Institute for Clinical Epidemiology and Biostatistics, University Hospital Basel, Basel, Basel-Stadt Switzerland; 4 Department of Clinical Epidemiology and Biostatistics, McMaster University, Hamilton, ON Canada

**Keywords:** Pro-Adrenomedullin, 10-year follow-up, Primary care, Outcome

## Abstract

**Background:**

Several studies found mid-regional pro-adrenomedullin (ProADM), the prohormone of the cardiovascular protein adrenomedullin, to be strongly associated with short-term mortality, mostly in the inpatient setting. We evaluated associations of ProADM levels with 10-year mortality in community-dwelling primary care patients with respiratory tract infections.

**Methods:**

This is a post-hoc analysis using clinical and biomarker data of 134 primary care patients with respiratory tract infections. ProADM was measured on admission and after 7 days in batch-analysis. 10-year follow-up data was collected by GP, patient and relative tracing through phone interviews. We calculated Cox regression models and area under the receiver operating characteristics curves to assess associations of ProADM with 10-year all-cause mortality.

**Results:**

During the 10-year follow-up 6% of included patients died. Median baseline ProADM blood levels (nmol/l) were significantly higher in non-survivors compared to survivors (0.5, IQR 0.4–1.3; vs. 0.2, IQR 0.1–0.5; *p* = 0.02) and showed a significant association with 10-year all-cause mortality in an age-adjusted cox regression model (HR: 2.5, 95%-CI: 1.0–6.1, *p* = 0.04). ProADM levels on day 7 showed similar results.

**Conclusions:**

This posthoc analysis found an association of elevated ProADM blood levels and 10-year all-cause mortality in a primary care cohort with respiratory tract infections. Due to the methodological limitations including incomplete data regarding follow-up information and biomarker measurement, this study warrants validation in future larger studies.

**Trial registration:**

Current Controlled Trials, SRCTN73182671

## Background

Prognostic blood biomarkers have generated clinical interest, resulting in therapeutic and diagnostic improvements [1]. A more personalized and targeted medicine offers opportunities to enhance treatment safety, efficacy as well as cost effectiveness. In this context, mid-regional pro-adrenomedullin (ProADM) provides prognostic information in various clinical settings and across different patient populations, predominantly suffering from cardiovascular and infectious diseases [2–8].

Adrenomedullin (ADM) is a 52-amino acid ringed peptide, belonging to the calcitonin peptide superfamily [9, 10]. It is produced ubiquitously by endothelial cells in cardiovascular, renal, pulmonary, cerebrovascular and endocrine tissues [11–13]. Studies reported several effects of ADM including vasodilatory, natriuretic, diuretic, anti-oxidative, anti-inflammatory, antimicrobial and metabolic effects [14–19]. Unfortunately, ADM measurement is challenging and not available outside the research setting. ProADM, the prohormone of ADM, however, is less biologically active, stable and commercially available [20].

Several studies found ProADM to be a strong and independent predictor of outcomes in patients with COPD [21], acute or chronic heart failure [22–26], community-acquired pneumonia [27, 28] and sepsis [29–32]. ProADM blood levels were also associated with morbidity after acute coronary syndrome [33–36], and predicted adverse outcomes in patients presenting to the emergency department (ED) with dyspnea [23, 37] or even nonspecific complaints [38]. Also, studies found association of ProADM with metabolic syndrome and its components, i.e. type 2 diabetes [39, 40].

Stratification of patients with lower respiratory infection based on a clinical algorithm including ProADM stratification tended to shorten length of hospital stay without an increase in adverse clinical outcome [41, 42].

In the setting of primary care, in smaller studies ProADM was associated with mortality in patients with urinary tract infections [43], type 2 diabetes [43–45] or heart failure [25, 46–49]. It is not known whether this marker helps to predict long-term risk, which could be useful for directing preventive measures. The aim of the current analysis was to investigate the ability of ProADM to predict long-term all-cause mortality and adverse outcome in a primary care cohort, visiting their general practitioner for respiratory infections.

## Methods

### Study design and setting

This is a post-hoc analysis investigating ProADM in a primary care cohort enrolled between December 2004 and April 2006 into the PARTI intervention trial (Procalcitonin-Guided Antibiotic Use vs. a Standard Approach for Acute Respiratory Tract Infections in Primary Care) [50, 51]. In brief, this randomized, multicenter, non-inferiority trial investigated the feasibility of a PCT-guided antibiotic therapy visiting their GP for a respiratory infection. The use of antibiotics was more or less strongly discouraged based on defined PCT-cutoff ranges. The standard approach followed evidence-based guidelines for use of antibiotics. The aim of the trial was to investigate safety and efficacy of using PCT levels to guide antibiotic therapy.

The initial study protocol, as well as the present follow-up trial, were approved by the local Ethics Committee of Basel (EKBB). Written informed consent was obtained from all participating physicians and patients.

### Selection of participants

Initially, all patients with the diagnosis of upper or lower ARTI and the physician’s intention to prescribe antibiotics were included. Exclusion criteria were antibiotic use within the previous 28 days, psychiatric disorders or inability to give written informed consent, not being available for follow-up, not being fluent in German, severe immunosuppression, cystic fibrosis, active tuberculosis, and the need for immediate hospitalization. In this analysis, we included only patients, of which the 10-year follow-up and either baseline or day 7 ProADM levels were available.

### Data collection and endpoints

The primary endpoint was defined as long-term 10-year all-cause mortality. Secondary outcomes were adverse outcomes including death, pulmonary embolism, and major adverse cardiac or cerebrovascular events (MACCE), which includes cardiac infarction, cardiac arrest, stroke and transient ischemic attack. In addition, we investigated new onset of diabetes within the follow-up period.

To verify outcomes, we performed follow-up telephone interviews with patients, relatives and/or general practitioners (GPs) 10 years after the baseline visit, using systematic questionnaires. Also, the register of death of the cantons Basel-Stadt and Basel-Land was consulted if no information about vital status was available.

### Analysis of blood biomarkers

Blood samples were collected in the primary care centre from each patient on admission and after 7 days in ethylendiaminetetraacetic (EDTA) tubes and sent by courier to the central Laboratory of the University Hospital Basel for measurement of PCT. Leftover blood samples were frozen and stored at −80° for the later measurement of prognostic markers.

ProADM serum values were determined using a sandwich immunoassay with a functional interassay precision of 20% and an assay sensitivity assessed as being 0.12 nmol/L (B.R.A.H.M.S. Sevadil® LIA; B.R.A.H.M.S. GmbH, Hennigsdorf, Germany) [20].

### Statistical analysis

We used STATA 12.1 (STATA Corp, College Station, TX; USA) and created univariable and bivariable (adjusting for age) as well as a multivariable model (adjusting for age, randomisation arm (PCT group), smoking history) cox regression analysis to calculate hazard ratios (HR) and area under the receiver operating characteristics curve (AUC) to investigate the predictive accuracy of Pro-ADM. We used a natural logarithm (base *e*) transformation of all biomarker data before entering into the statistical models to approximate normal distribution. Therefore, HR corresponds to a 2.72-fold increase in log transformed biomarker levels. To illustrate the predictive power we used Kaplan-Meier plots for proportion of survivors and proportion of events by quartiles of biomarker levels. Log-rank tests were performed to compare quartiles. *P*-values of <0.05 were considered to indicate statistical significance.

## Results

### Patient population

The initial cohort included 458 adult patients with an ARTI, of which 167 (36.5%) had ProADM blood samples available, while the other patients had not enough leftover sample for measurement of ProADM. A total of 134 (80.2%) of these patients could be contacted to assess long-term outcomes between April and August 2015 and were, thus, included in the final analysis. For 291 (63.5%) patients of the initial cohort no data concerning ProADM blood levels was available due to missing blood tube.

Table 1 shows baseline characteristics of the overall cohort as well as stratified by survival status. The median age was 42.0 years and 32.8% of patients were male. Median ProADM blood levels on admission were 0.3 nmol/l and 0.2 nmol/l on day 7. According to survival status, there were significant differences in age, comorbidities (chronic obstructive pulmonary disease), initial clinical classification of type of respiratory infection and nicotine consumption (pack-years). A comparison between the initial cohort and the patients available for 10-year follow-up is presented in Table 5 (see Appendix).

**Table 1:** Patient characteristics at baseline and after 10 years, stratified by vital status

Characteristics	Included cohort	Survivors	Nonsurvivors	*p-value*
	*N* = 134	*N* = 126	*N* = 8	
Demographic characteristics				
Age median (IQR)	42.0 (30.0, 57.0)	41.0 (29.0, 55.0)	73.5 (67.5, 82.5)	*<0.001*
Male, No. (%)	44 (32.8)	39 (31.0)	5 (62.5)	*0.065*
Cardiovascular risk factors				
Smoker or former smoker (SD)	35 (26.1)	33 (26.2)	2 (25.0)	*0.94*
Pack years median (IQR)	12.0 (2.0, 20.0)	10.0 (2.0, 20.0)	45.0 (40.0, 50.0)	*0.043*
Positive family history (%)	13 (9.8)	13 (10.3)	0 (0.0, 0.0)	*0.41*
Comorbidities	21 (15.7)	18 (14.3)	3 (37.5)	
Arterial hypertension (%)	13 (9.7)	12 (9.5)	1 (12.5)	*0.78*
Dyslipoproteinemia (%)	9 (6.7)	8 (6.3)	1 (12.5)	*0.5*
Diabetes mellitus (%)	3 (2.2)	3 (2.4)	0 (0.0)	*0.66*
COPD (%)	3 (2.2)	2 (1.6)	1 (12.5)	*0.043*
Initial clinical condition				
Lower ARTI (%)	75 (56.0)	68 (54.0)	7 (87.5)	*0.064*
Upper ARTI (%)	59 (44.0)	58 (46.0)	1 (12.5)	*0.064*
ProADM at baseline (pmol/L) N = 130		*N* = 122	*N* = 8	
median (IQR)	0.3 (0.1,0.5)	0.2 (0.1, 0.5)	0.5 (0.4, 1.3)	*0.022*
mean (SD)	0.4 (0.4)	0.3 (0.3)	0.9 (0.8)	*<0.001*
ProADM at day 7 (pmol/L)	*N* = 123	*N* = 116	*N* = 7	
median (IQR)	0.2 (0.1,0.4)	0.2 (0.1,0.4)	0.4 (0.2, 0.7)	*0.08*
mean (SD)	0.3 (0.2)	0.3 (0.2)	0.5 (0.5)	*0.012*

Data are presented as median (IQR), mean (SD) or % (no.). *p* values are statistically significant at *p* < 0.05. *IQR* Interquartile range (25th–75th percentiles), *SD* Standard deviation, *CV* cardiovascular, *COPD* Chronic obstructive lung disease, *ARTI* Acute respiratory tract infection. Comorbidities were identified based on medical record of GP, patient report, or both

A similar 10-year post-hoc analysis of the prognostic biomarkers copeptin and MR-proANP, based on the same blood samples from the PARTI trial, has been published recently and showed that copeptin as well as MR-proANP were associated with 10-year all-cause mortality [52, 53].

### Primary outcome: 10-year all-cause mortality

During the follow-up of 10 years (mean: 9.5 years), mortality was 6% (*n* = 8 patients). Median admission ProADM blood levels (nmol/l) were significantly higher in non-survivors compared to survivors (0.5, IQR 0.4–1.3 (*n* = 8); vs. 0.2, IQR 0.1–0.5 (*n* = 122); *p* = 0.02). Similar results were found at day 7 (0.4, IQR 0.2–0.7 (*n* = 7); vs. 0.2, IQR 0.1–0.4 (*n* = 116); *p* = 0.08).

Initial ProADM level showed a strong association with 10-year all-cause mortality in a univariable cox regression model (HR: 4.2, 95%-CI: 1.5, 11.5, *p* = 0.006). The result remained significant when adjusted for age (adjusted HR: 2.5, 95%-CI: 1.0–6.1, *p* = 0.043). There was no significant effect modification by randomisation arm (PCT group) or a positive history of smoking. Day seven ProADM levels showed in the univariable model a hazard ratio of borderline significance (HR: 2.7, 95%-CI: 1.0–7.4, *p* = 0.06).

The areas under the receiver operating curve (AUC) suggest fair accuracies of ProADM levels at baseline and on day seven (AUC: 0.74, 95%-CI: 0.54–0.94 and AUC: 0.70, 95%-CI: 0.48, 0.91).

**Fig. 1:**
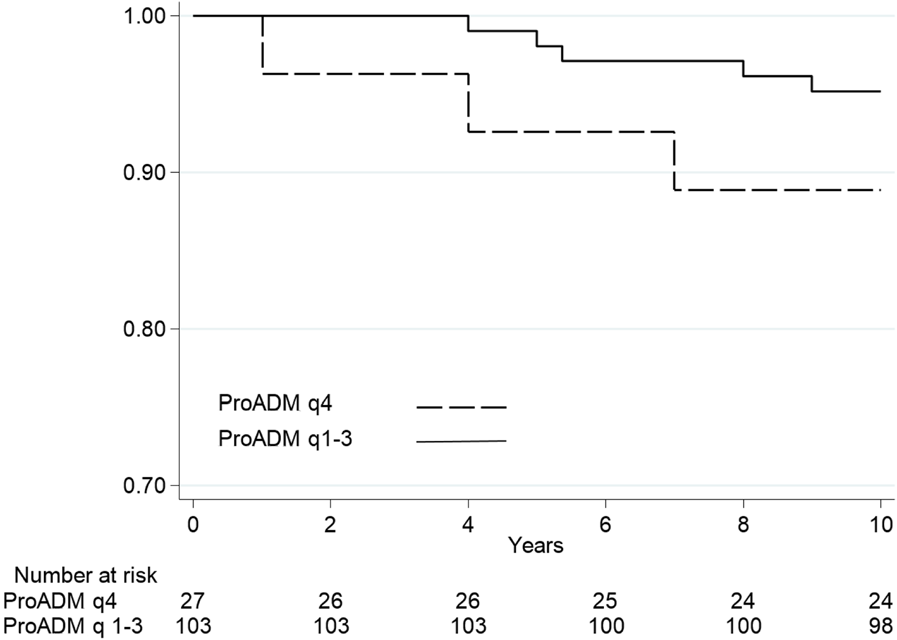
Quartiles of baseline ProADM and 10-year Kaplan-Meier survival. Plots showing the association between endpoint and ProADM quartiles, 4th quartile versus 1st – 3rd quartiles

**Fig. 2:**
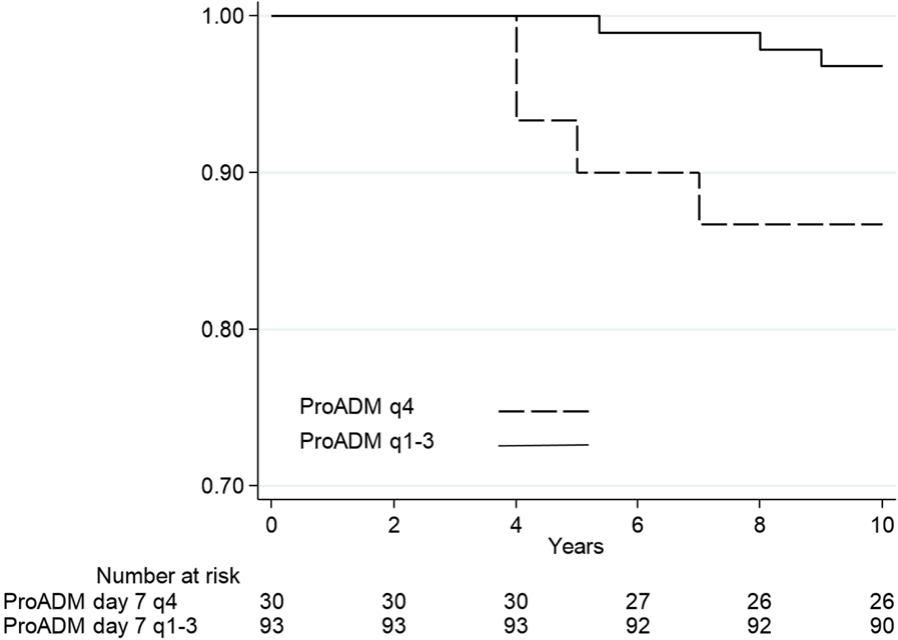
Quartiles of follow-up ProADM and 10-year Kaplan-Meier survival. Plots showing the association between endpoint and ProADM quartiles, 4th quartile versus 1st – 3rd quartiles

We generated Kaplan-Meier curves to visualize the difference of survival between the highest quartile and lower three quartiles on admission and on day 7 (Figs. 1 and 2). The log-rank-test showed no significant increase in mortality when comparing the highest ProADM quartile to quartiles 1 to 3.

### Secondary outcomes

During the 10-year follow-up, 9.7% of patients (*n* = 13) had an adverse outcome event including death, pulmonary embolism, and major adverse cardiac or cerebrovascular events (MACCE). At baseline, median ProADM levels (nmol/L) showed no statistically significant difference between patients with or without adverse outcome event (0.4, IQR 0.2–0.5; vs. 0.3, IQR 0.1–0.5; *p* = 0.15).

Cox regression models found borderline significant associations between initial and ProADM levels and adverse outcome, with an AUC of 0.62 (Table 2). When adjusted for age (HR: 1.4, 95%-CI: 0.8 to 2.5), *p* = 0.273), there was no significant association found. As well, there was no significant effect modification by randomisation arm (PCT group) or a positive history of smoking.

**Table 2:** Association between ProADM blood levels at baseline and day 7 and 10-year outcomes

baseline	all-cause mortality	adverse outcome
Unadjusted HR	4.2 (1.5 to 11.5), *p* = 0.006	1.9 (1.0 to 3.7), *p* = 0.054
age-adjusted HR	2.5 (1.0 to 6.1), *p* = 0.043	1.4 (0.8 to 2.5), *p* = 0.273
age&group adjusted HR	2.7 (1.0 to 7.1), *p* = 0.047	1.5 (0.8 to 2.8), *p* = 0.234
age&group&smoking adjusted HR	2.7 (1.0 to 7.1), *p* = 0.051	1.4 (0.7 to 2.7), *p* = 0.314
AUC	0.74 (0.54 to 0.94)	0.62 (0.45 to 0.80)
day 7		
Unadjusted HR	2.7 (1.0 to 7.4), *p* = 0.055	1.8 (0.9 to 3.7), *p* = 0.108
age-adjusted HR	1.7 (0.7 to 4.1), *p* = 0.282	1.2 (0.6 to 2.5), *p* = 0.547
age&group adjusted HR	1.8 (0.7 to 4.6), *p* = 0.245	1.3 (0.6 to 2.6), *p* = 0.469
age&group&smoking adjusted HR	1.7 (0.7 to 4.6), *p* = 0.262	1.2 (0.6 to 2.6), *p* = 0.592
AUC	0.70 (0.48 to 0.92)	0.63 (0.45 to 0.81)

Data for univariable, bivariable and multivariable analyses are given as HR (95% CI) and *p* value. Data regarding prognostic analysis are given as AUC (95%CI). A higher AUC reflects greater accuracy: 0.5, the null value, indicates coin-tossaccuracy, while 1.0, the maximum value, indicates 100% accuracy. *P* values in bold type are statistically significant at *p* < 0.05

Univariable model includes: natural logarithmic value of ProADM blood concentrations

Bivariable model includes: natural logarithmic value of ProADM blood concentrations and patient age

Significance tests for follow-up ProADM levels showed similar results.

**Fig. 3:**
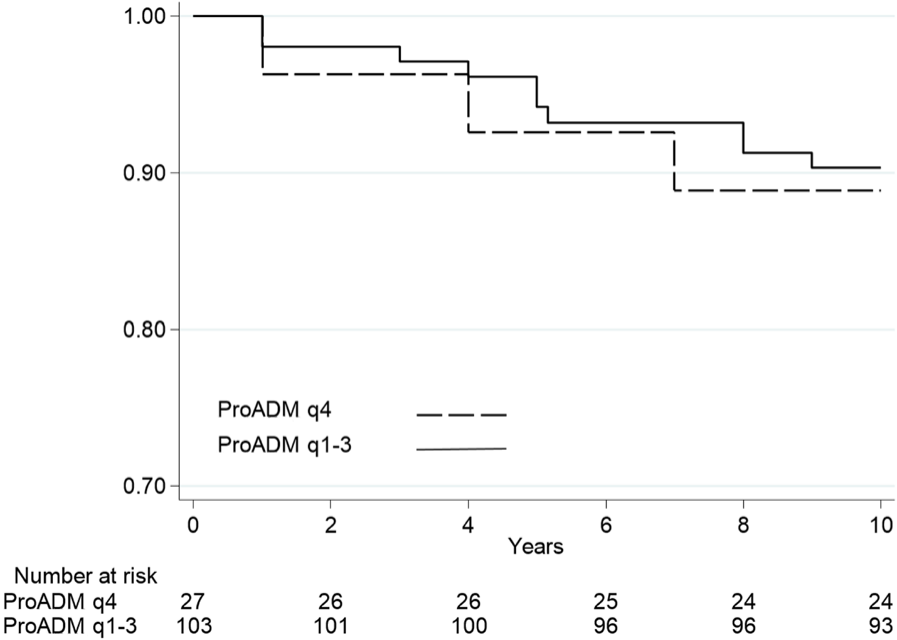
Quartiles of baseline ProADM and incidence of adverse outcome. Plots showing the association between endpoint and ProADM quartiles, 4th quartile versus 1st – 3rd quartiles

**Fig. 4:**
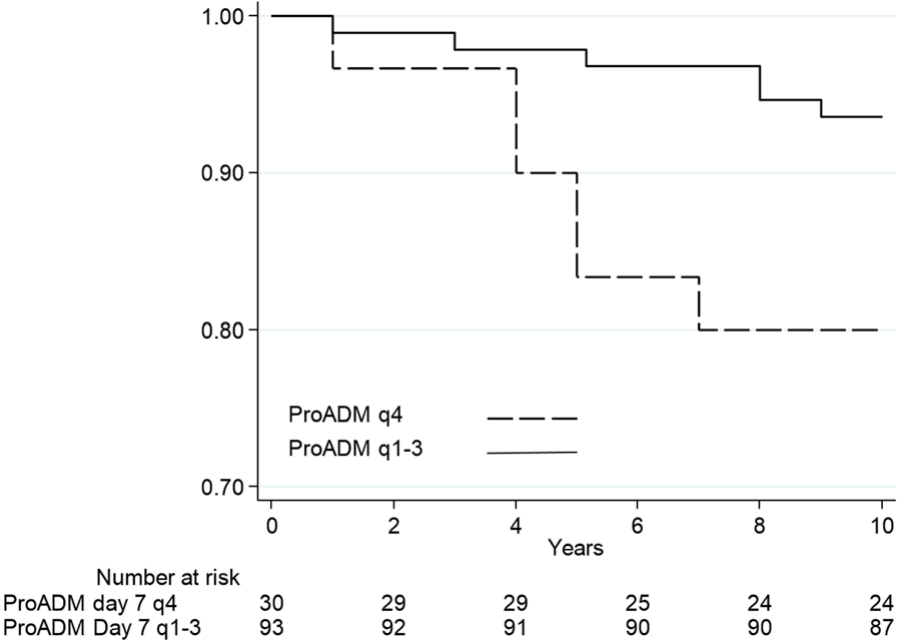
Quartiles of follow-up ProADM and incidence of adverse outcome. Plots showing the association between endpoint and ProADM quartiles, 4th quartile versus 1st – 3rd quartiles

Kaplan-Meier curves (Figs. 3 and 4), with patients stratified based on ProADM quartiles, illustrate the slightly higher event rates in patients in the highest ProADM quartile.

Further, no association between ProADM and MACCE or new-onset diabetes mellitus was found (see Appendix).

## Discussion

Within this prospective, observational 10-year follow-up study of a small cohort of community-dwelling ARTI patients, we found ProADM on admission and after 7 days to be an age-independent predictor for all-cause mortality. No significant associations of ProADM and secondary outcomes were found, namely adverse outcome, MACCE and new onset of diabetes.

Our results are in-line with previous research reporting associations of ProADM and short-term mortality in inpatients [2–4, 21–38] as well as in primary care populations [25, 43–49]. Importantly, also in long-term follow-up over 10 years the prognostic accuracy remained stable over time. Thus, based on this study and previous research, ProADM is a short- and long-term valid prognostic marker for patients from the community who may benefit from preventive measures. Our study suggests that ProADM should be evaluated in future long-term studies in outpatients assessing the accuracy of clinical scores (e.g., Pneumonia Severity Index, CURB65 (confusion, uremia, respiratory rate, blood pressure, age at least 65 years) score or Framingham score) in combination with novel markers to predict long-term outcome in this setting and direct individual use of medication or even hospital admission.

Although there is no clear understanding why an increase in ProADM points to increased mortality risk, existing data suggests that elevated ProADM levels reflect disease severity and endothelial and cardiovascular dysfunction [11–19]. Also, higher ADM levels increases cardiac output, induces hypotension and vasodilation, and increases glomerular filtration rate and fractional sodium excretion [10, 19, 54], thereby inducing a reduction in cardiac pre- and afterload [20]. Thus, the involvement of ADM in several pathological disease states and comorbidities might explain the associations found in this and previous studies.

Median ProADM blood levels in our outpatient cohort were 0.3 nmol/l on admission and 0.2 nmol/l on day 7 and thus significantly lower compared to other hospitalized patient cohorts. The AtheroGene study found median ProADM levels of around 0.5 and 0.6 nmol/L in patients with stable angina and acute coronary syndrome [35]. The LAMP study reported median concentration of 0.73 nmol/L in patients with myocardial infarction [36], while the GISSI study found a median ProADM concentration of 0.75 nmol/L in patients with chronic heart failure [26]. Analysis of a presumably healthy subset in a large outpatient cohort (*n* = 5258) lead to a reference interval of 0.23—0.64 nmol/L [18, 55]. These differences demonstrate that levels of ProADM need to be adapted to the specific clinical setting to be interpreted in a meaningful way.

Interestingly, although several previous studies have suggested that ProADM was also associated with other adverse outcomes in addition to all-cause mortality [26, 27, 33–36, 44–46], we did not find such statistically significant association with the incidence of our secondary combined endpoint including pulmonary embolism and MACCE. Also, in contrast to another study [39], our analysis did not find an association of ProADM with new onset diabetes mellitus. This may be due to the small number of events in our generally healthy population with a low burden of comorbidities and thus low power of our analysis. Further, the respiratory infection of patients during the initial trial may have had an influence on ProADM levels. Thus, similar to lipid levels, [56] this marker may be best analyzed during stable conditions for the purpose of long-term risk assessment.

The main strengths of this study include the 10 years of follow-up, the participation of multiple GP practices, and the community sample of patients with ARTI of different severity representative for patients mainly treated in primary care. Nonetheless, we are aware of several limitations. First, this is a secondary analysis of a previous trial and baseline risk assessment is incomplete as is the availability of ProADM levels in the cohort. For 291 (63.5%) patients no data concerning ProADM blood levels was available, because blood sampling was only done in a sub fraction of the overall cohort during a certain time period. Selection bias is thus possible. Second, due to the long follow-up period, a recall bias has to be considered. Further, no information was available on the cause of death, when patients were tracked through the register of deaths. Third, our sample was small and we observed only few events for the analysis of the relationship between ProADM levels and adverse outcomes.

## Conclusion

This posthoc analysis found an association of elevated ProADM blood levels and 10-year all-cause mortality in a primary care cohort with respiratory tract infections. Due to the methodological limitations including incomplete data regarding follow-up information and biomarker measurement, this study warrants validation in future larger studies. If validated, ProADM may help to risk stratify patients and thereby allows to improve allocation of health care resources and preventive measures.

## Appendix

**Table 3:** Association between ProADM blood levels at baseline and day 7 and 10-year outcomes

baseline	New-onset DM	MACCE
Unadjusted HR	0.9 (0.3 to 2.5), *p* = 0.773	0.8 (0.2 to 2.4), *p* = 0.634
age-adjusted HR	0.8 (0.3 to 2.3), *p* = 0.641	0.7 (0.2 to 2.2), *p* = 0.54
AUC	0.50 (0.12 to 0.87)	0.42 (0.11 to 0.73)
day 7		
Unadjusted HR	1.2 (0.4 to 4.1), *p* = 0.717	1.1 (0.3 to 3.6), *p* = 0.873
age-adjusted HR	1.1 (0.3 to 3.7), *p* = 0.884	0.9 (0.3 to 2.8), *p* = 0.808
AUC	0.56 (0.16 to 0.97)	0.53 (0.15 to 0.91)

Data for univariable and bivariable analyses are given as HR (95% CI) and *p* value. Data regarding prognostic analysis are given as AUC (95%CI). A higher AUC reflects greater accuracy: 0.5, the null value, indicates coin-tossaccuracy, while 1.0, the maximum value, indicates 100% accuracy. *P* values in bold type are statistically significant at *p* < 0.05

Univariable model includes: natural logarithmic value of ProADM blood concentrations

Bivariable model includes: natural logarithmic value of ProADM blood concentrations and patient age

*MACCE* Major cardiovascular and cerebrovascular events, *New-onset DM*, New- onset diabetes mellitus

**Table 4:** Association between ProADM blood levels at baseline and day 7 and diabetes mellitus at time of follow-up

baseline	diabetes mellitus
Unadjusted OR	0.8 (0.3 to 2.0), *p* = 0.623
age-adjusted OR	0.7 (0.3 to 1.8), *p* = 0.493
AUC	0.45 (0.20 to 0.71)
day 7	
Unadjusted OR	0.7 (0.3 to 1.7), *p* = 0.437
age-adjusted OR	0.6 (0.2 to 1.5), *p* = 0.278
AUC	0.41 (0.14 to 0.69)

Data for univariable and bivariable analyses are given as odds ratio (OR) (95% CI) and *p* value

Data regarding prognostic analysis are given as AUC (95%CI)

A higher AUC reflects greater accuracy: 0.5, the null value, indicates coin-tossaccuracy, while 1.0, the maximum value, indicates 100% accuracy

*P* values in bold type are statistically significant at *p* < 0.05

Univariable model includes: natural logarithmic value of ProADM blood concentrations

Bivariable model includes: natural logarithmic value of ProADM blood concentrations and patient age

Diabetes mellitus: patients with DM at baseline and new- onset DM

**Table 5:** Patient characteristics at baseline, stratified by availability for follow-up analysis

Characteristics	Entire cohort	Excluded cohort^a^	Included cohort	p-value^b^
	*N* = 458	*N* = 324	*N* = 134	
Demographic characteristics				
Age median (IQR)	45 (34, 62)	47 (36, 63)	42 (30, 57)	0.022
Male, No. (%)	185 (40.4%)	141 (43.5%)	44 (32.8%)	0.034
Initial clinical condition				
Lower ARTI (%)	248 (54.1%)	173 (53.4%)	75 (56.0%)	0.61
Upper ARTI (%)	210 (45.9%)	151 (46.6%)	59 (44.0%)	0.61
ProADM at baseline (pmol/L)	*N* = 163	*N* = 33	*N* = 130	
median (IQR)	.3 (.1, .5)	.4 (.2, .5)	.3 (.1, .5)	0.022
mean (SD)	.4 (.3)	.4 (.3)	.4 (.4)	0.29
ProADM at day 7 (pmol/L)	*N* = 151	*N* = 28	*N* = 123	
median (IQR)	.2 (.1, .4)	.4 (.2, .5)	.2 (.1, .4)	0.012
mean (SD)	.3 (.3)	.4 (.3)	.3 (.3)	0.033

Data are presented as median (IQR), mean (SD) or % (no.). *p* values are statistically significant at *p* < 0.05

*IQR* Interquartile range (25th–75th percentiles), *SD* Standard deviation, *ARTI* Acute respiratory tract infection

^a^No ProADM blood levels measured or lost to follow-up, ^b^ Included cohort versus excluded cohort

## Abbreviations

ARTI: acute respiratory tract infection
AUC: area under the receiver operating characteristic curve
CI: confidence interval
COPD: chronic obstructive pulmonary disease
CV: cardiovascular
DM: diabetes mellitus
ED: emergency department
EKBB: Ethics Committee of Basel (Switzerland)
GP: general practitioner
HR: hazard ratio
IQR: interquartile range (25th–75th percentiles)
MACCE: major adverse cardiac and cerebrovascular event
NPV: negative predictive value
PCT: procalcitonin
PPV: positive predictive value
ProADM: MR-pro-Adrenomedullin
SD: standard deviation
